# Impact of NQO1 dysregulation in CNS disorders

**DOI:** 10.1186/s12967-023-04802-3

**Published:** 2024-01-02

**Authors:** Li Yuhan, Maryam Khaleghi Ghadiri, Ali Gorji

**Affiliations:** 1 Epilepsy Research Center, Münster University, Münster, Germany; 2 Department of Breast Surgery, Xinhua Hospital Affiliated to Shanghai Jiao Tong University School of Medicine, Shanghai, China; 3 Department of Neurosurgery, Münster University, Münster, Germany; 4 Shefa Neuroscience Research Center, Khatam Alanbia Hospital, Tehran, Iran; 5 Neuroscience Research Center, Mashhad University of Medical Sciences, Mashhad, Iran

**Keywords:** Brain, CNS disorders, Inflammation, Neurodegeneration, Neuronal excitability, NQO1, Biomarker

## Abstract

NAD(P)H Quinone Dehydrogenase 1 (NQO1) plays a pivotal role in the regulation of neuronal function and synaptic plasticity, cellular adaptation to oxidative stress, neuroinflammatory and degenerative processes, and tumorigenesis in the central nervous system (CNS). Impairment of the NQO1 activity in the CNS can result in abnormal neurotransmitter release and clearance, increased oxidative stress, and aggravated cellular injury/death. Furthermore, it can cause disturbances in neural circuit function and synaptic neurotransmission. The abnormalities of NQO1 enzyme activity have been linked to the pathophysiological mechanisms of multiple neurological disorders, including Parkinson's disease, Alzheimer's disease, epilepsy, multiple sclerosis, cerebrovascular disease, traumatic brain injury, and brain malignancy. NQO1 contributes to various dimensions of tumorigenesis and treatment response in various brain tumors. The precise mechanisms through which abnormalities in NQO1 function contribute to these neurological disorders continue to be a subject of ongoing research. Building upon the existing knowledge, the present study reviews current investigations describing the role of NQO1 dysregulations in various neurological disorders. This study emphasizes the potential of NQO1 as a biomarker in diagnostic and prognostic approaches, as well as its suitability as a target for drug development strategies in neurological disorders.

## Introduction

NAD(P)H quinone dehydrogenase 1 (NQO1) is an enzyme involved in cellular detoxification and protection against oxidative stress. NQO1belongs to the quinone oxidoreductase family and is found in different tissues, including the lung, thyroid, colon, heart, kidney, liver, cornea, and lens, as well as the peripheral and central nervous systems (CNS) [1]. Disruption of the oxidant/antioxidant balance in the CNS exerts significant effects on various physiological processes and contributes to the evolution of several acute and chronic neurological disorders. NQO1 participates in the cell protection process against unwanted oxidative damage and maintains cellular homeostasis through the reduction of free radicals and detoxifying deleterious quinones as well as the modulation of antioxidant genes [2, 3].

Furthermore, NQO1 exerts both direct and indirect modulation over the activity of various signaling pathways, including those that influence cell proliferation [4, 5], apoptosis [6], and neuroinflammation [7]. In the CNS, NQO1 governs the inflammatory responses via different mechanisms, including the modulation of particular pro-inflammatory cytokines and activation of CNS immune cells, like astrocytes and microglial cells [8]. NQO1 contributes to mitigating inflammation in various physiological and pathological conditions through the reduction of oxidative damage and quenching of oxidative stresses (ROS). On the other hand, several factors impact the expression and function of NQO1 in the CNS, including oxidative stress, hormonal signaling pathways, and epigenetic modifications.

Different polymorphisms in the NQO1 gene influence the translation of the NQO1 protein and contribute to the occurrence of various neurological diseases. For instance, the C609T polymorphism exerts an influence on various neurodegenerative and neuroinflammatory disorders, such as Alzheimer´s disease (AD) and multiple sclerosis (MS) [9, 10]. Here, we first summarized the current knowledge of the modulatory mechanisms of NQO1 in the CNS. Moreover, we provided a comprehensive review of the current experimental and clinical knowledge of the potential prognostic, diagnostic, and therapeutic roles of the NQO1 in different neurological disorders. Finally, we discussed the challenges in designing and developing novel substances that target NQO1 for diagnostic, preventive, and therapeutic purposes in neurological disorders. Understanding the function of NQO1 and its modulatory role in the CNS is crucial for revealing its potential therapeutic implications in various neurological disorders [11].

## General aspects of NQO1

### Structure

NQO1 is a flavoprotein that acts as an enzyme and is composed of a NAD(P)H-binding domain and a quinone-binding domain that is encoded by the NQO1 gene [12, 13]. This ubiquitous cytosolic enzyme consists of two uniform subunits, with a dimer molecular weight of about 52 kDa. The exact molecular weight of NQO1 may vary slightly depending on different factors, like tissue source, post-translational modifications, and measurement techniques. The NQO1 gene is located on chromosome 16q22.1 and the sequence of the NQO1 gene can vary between individuals due to genetic polymorphisms. The human NQO1 gene is approximately 20 kb and composed of 6 exons that are interspersed by 5 introns [14]. NQO1 tightly binds flavin adenine dinucleotide (FAD; as a cofactor), which plays an important role in the stability and activity of the enzyme [15]. FAD is involved in the formation of homodimers and plays a role in the reduction of two-electron of quinones to form hydroquinones [16]. NQO1 inhibitors exert their effects through the binding to the NAD(P)H and consequently obstructing the transfer of electrons to FAD [17], particularly through modulation of Tyr 128 and Phe 232 on the surface of the active site of the enzyme [18]. Under physiological conditions, NQO1 is primarily a cytosolic enzyme, but a smaller quantity of NQO1 has been also detected in the nucleus [19].

### Functions

Various forms of stress, including oxidative stress, stimulate the bioactivity of NQO1, which plays a crucial role in safeguarding cells against damage and preventing cell death. NQO1 is a cytoprotective antioxidant substance that regulates ROS. NQO1 reduces toxic quinines to form hydroquinone via a single-step two-electron reduction reaction by bypassing the formation of the highly reactive semiquinone [20]. Semiquinone produces ROS through the redox cycle, and the accumulation of ROS results in cell damage and cell death [21]. Therefore, NOQ1 degrades quinones and prevents massive accumulation of ROS and DNA damage from oxidative insults. Experiments using purified recombinant human NQO1 and cancerous as well as non-cancerous cells have shown that NQO1 can also use the electrons provided by NADH or NAD(P)H to react with quinone compounds, directly donating quinone double electrons for the reduction reaction, avoiding the formation of semiquinone, a metabolic intermediate that is carcinogenic and teratogenic, and replacing it with a direct reduction to hydroquinones, and then convert hydroquinones into water-soluble compounds to be excreted from the body, reducing the cytotoxic reaction [22–25]. NQO1 plays a role in the maintenance of several endogenous antioxidants. Studies on the cellular distribution pattern of NQO1 in MS brains have revealed that alterations in NQO1 levels can play a role as a marker for cellular oxidative stress [26]. In experiments with purified human NQO1, it has been shown that NQO1 interacts with α-tocopherol quinone, generated at slow levels during the antioxidant action of vitamin E, to form α-tocopherol hydroquinone and protects against lipid peroxidation of the cell membrane [22, 23]. At relatively high levels of NQO1 in the cells and low values of superoxide dismutase, NQO1 restores α-tocopherol to its hydroquinone form to protect the membranes against lipid peroxidation [27, 28]. Furthermore, studies on fractionated mouse liver extracts and human colon carcinoma cells have revealed that NQO1 can directly reduce superoxide and modulate the degradation of some proteins, such as P53 [19, 29]. NQO1 also indirectly reduces ROS via the changes of different antioxidants, such as vitamin E and coenzyme Q10 [3]. Besides, NQO1 is inhibited by several anticoagulants, such as dicumarol, and strongly decreases vitamin K metabolism [30]. Moreover, investigations on various human cancer cells revealed that NQO1 directly interferes with the unstructured DNA-binding domain of c-Fos and leads to the upregulation of cyclin-dependent kinase subunit-1 and the modulation of the cell-cycle progression at the G2/M phase [5]. NQO1 catalyzes the reduction of membrane-bound coenzyme Q (a ubiquinone analog) to their ubiquinol forms in liposomes and protects membrane phospholipids from oxidative damage [31]. Unrelated to its enzymatic activity, there are cytoprotective roles for NQO1 through several mechanisms, such as the activation of apoptosis. NQO1 binds the tumor suppressor protein p53 and enhances its stability through the inhibition of proteasomal degradation [32]. In addition, the evaluation of vascular endothelial and smooth muscle cell redox signaling revealed that the nuclear factor erythroid 2-related factor 2 (Nrf2)-NQO1 pathway controls cell injury/death through the maintaining of lipid peroxidation and the synthesis of 4-hydroxynonenal, a lipid hydroperoxide end product [33]. It has been demonstrated that the suppression of receptor-interacting serine/threonine-protein kinase 1 inhibits cell death pathways (such as necroptosis and apoptosis) and leads to the suppression of NQO1 activity and NQO1-dependent cell death in various cell lines [34].

Moreover, NQO1 has been shown to modulate the redox state of various transcription factors, such as p53, AP-1, and Nrf2, thereby influencing the expression of diverse genes implicated in cell cycle control, apoptosis, and antioxidant defense. NQO1 has been shown to stabilize p53, a tumor suppressor protein, through a redox mechanism involving the oxidation of NAD(P)H [32, 35]. The interaction between NQO1 and p53 can vary among different cell types, potentially due to the influence of other p53-interacting proteins that dominate in particular cellular contexts [36]. Furthermore, the effect of NQO1 on P53 has been evaluated in in both endothelial cells and various cancer cell types, such as human colon carcinoma cell. NQO1 protects p53 and some other proteins from protease degradation through protein–protein bidirectional interactions [35, 37, 38]. Furthermore, NQO1 modulates the stability of other proteins involved in regulating cell growth and cell death, such as p73 and p33ING1b [3, 5, 28, 39–43]. C/EBPβ (CCAAT/enhancer binding protein β) is a group of transcription factors that play a multifaceted role in diverse cellular processes, such as proliferation, differentiation, and immune response regulation. Studies on different in vitro experimental models suggest that NQO1 and C/EBP may exert bidirectional regulatory interactions on each other and modulate cell proliferation and differentiation in some neurological disorders [44]. NQO1 also interferes with Nrf2 and the enhancer sequence of the antioxidant response element (ARE) [45–50].

NQO1 interaction with mRNA can play a role in cell defense against ROS, malignancy, and metabolic stress by alterations in gene expression, protein synthesis, and cellular function [51]. Using cellular and animal models of cardiac hypertrophy, it has been shown that NQO1 can modulate the stability of mRNA by binding to specific sequences within the mRNA molecule [52]. Furthermore, several cell culture investigations have shown that NQO1 can directly influence the translation of mRNA into protein by interfering with the ribosome or other components of the translation machinery [53, 54]. A meta-analysis of NQO1 and ribosome subunit gene expression in brain specimens from patients with schizophrenia and controls (overall 511 samples) revealed a positive correlation between NQO1 and upregulated ribosome subunit genes, suggesting that the upregulation of ribosome subunits may lead to altered mRNA translation and disruptions in neuronal network functions [55].

### Polymorphism

The locus of the human NQO1 gene exhibits high polymorphism, and different variations have been reported, particularly in the promoter area [56]. NQO1 polymorphism can lead to decreased enzyme activity and stability, leading to increased sensitivity to oxidative stress. Multiple polymorphisms in the NQO1 gene have been associated with altered enzyme activity, which has implications for various health conditions, including cancer, cardiovascular diseases, and neurological disorders. The relationship between NQO1 polymorphisms and neurological disorders is complex and depends on the specific polymorphism and the genetic background of patients [57]. NQO1 polymorphisms are associated with an increased risk of some neurological disorders, such as MS and AD, likely due to structural and functional alterations of NQO1 and disruption in the cellular antioxidant defence [58]. Two main polymorphisms of NQO1 in humans are C609T and C465T. The most influential NQO1 polymorphism is the C609T polymorphism (known also as polymorphism P187S or NQO1*2), which significantly increases the susceptibility for developing various malignancies, such as hepatocellular, bladder, and pulmonary cancers [24, 25, 39, 59–70]. A meta-analysis involving 92 studies, which included 21,178 patients with various types of cancer and 25,157 healthy controls, indicates the implication of C609T polymorphism of the NQO1 gene as a genetic risk factor in different cancers [71]. The C609T polymorphism leads to a proline-to-serine change at codon 187 of the protein, which markedly decreases quinine reductase activity and enzyme stability [70]. NQO1 polymorphism C609T is also associated with enhanced risk of different neurological diseases, such as AD, MS, and Parkinson's disease (PD), presumably via destabilization of FAD affinity and enzyme stability [72]. In a meta-analysis of five case–control investigations involving 735 patients with AD and 828 controls, an association between the NQO1 C609T polymorphism and AD in Chinese populations has been demonstrated [9]. Furthermore, the C609T polymorphism impacts cell redox metabolism as well as cell sensitivity to different therapeutic approaches [73]. This polymorphism influences the response to chemotherapy and radiotherapy in patients with cancer and could be used as a potential prognostic biomarker for cancer therapy resistance [74]. Furthermore, the NQO1 C465T polymorphism (known also as NQO1*3) is a single-nucleotide alteration at nucleotide 465, where a cytosine is replaced by a thymine base, and leads to a marked reduction of enzyme activity [75]. This polymorphism is also associated with an increase in the risk for various cancers [76, 77]. For instance, a meta-analysis that included 28 relevant studies involving 5,953 patients with acute leukemia and 8,667 controls has shown an increased risk of cancer for carriers of the NQO1 C609T polymorphism [77]. The 3423G is another NQO1 polymorphism, which has been accompanied by decreased enzyme activity and enhanced risk of cancer [78]. The frequency and distribution of different NQO1 polymorphisms vary among various ethnic and racial populations [71, 79, 80]. Considering genotype–phenotype correlations in the multifunctionality of the NQO1 protein, genetic diversity, and the influence of post-translational modifications, NQO1 emerges as an exemplary model of a disease-associated antioxidant protein [81]. Furthermore, various therapies designed to correct the stability and dynamics of NQO1 polymorphic proteins and to regulate intracellular factors leading to loss-of-function are proposed as promising approaches for achieving better outcomes [17]. Examining epigenetic modifications on the NQO1 gene and their potential interactions with these polymorphisms could represent an innovative research approach [82].

## NQO1: the regulatory pathways

### Nrf2‑KEAP1‑ARE‑NQO1 signaling pathway

The NQO1-Kelch-like ECH-associated protein1 (KEAP1)-Nrf2-ARE signaling pathway involves a series of molecular interactions that contribute to the adaptive cellular stress response, particularly to chemicals that are electrophilic or oxidative in nature [83]. Increasing evidence suggests the protective role of the Nrf2-ARE signal pathway in central nervous diseases mediated by heme oxygenase-1 (HO-1) and NQO1 [84, 85]. The activation of the NQO1 gene occurs in conjunction with other Nrf2-induced detoxifying enzyme genes, including glutathione S-transferase (GST) and HO-1. This activation is triggered by various stimuli, such as free radical scavengers, hypoxic insults, ionizing electromagnetic radiation, chemical substances, heat shock, and heavy metals [86, 87]. Activation of the transcription factor Nrf2 has been shown to provide protection against the harmful effect of excessive oxidative stress in various pathological conditions [1]. Moreover, Nrf2 activation enhances the availability of substrates for mitochondrial function and promotes adenosine triphosphate (ATP) production, which can have a cell-protective effect [88, 89]. Overexpression of Nrf2 significantly enhances the expression of NQO1 and exerts a strong neuroprotective effect on neurons and neuroglial cells [90].

**Fig. 1:**
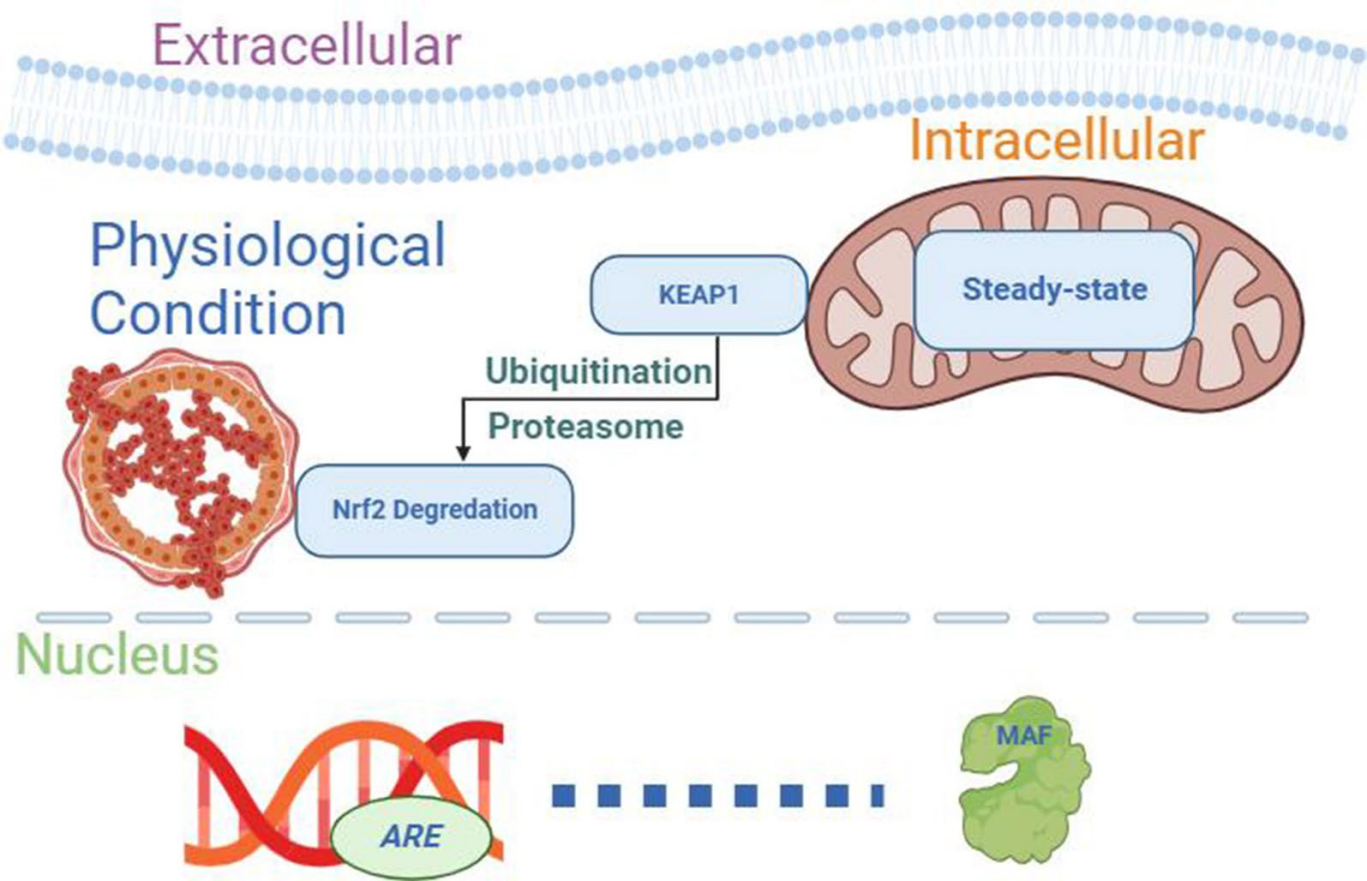
Schematic depiction of the induction and functions of the Nrf2-NQO1 pathway in physiological conditions. In a steady-state condition, erythroid 2-related factor 2 (Nrf2) is sequestered within the cytosol by the repressor protein Kelch-like ECH-associated protein1 (KEAP1). This is crucial for KEAP1 to remain unaffected by external factors, preserving its original conformation. This stability ensures the natural degradation of Nrf2 and prevents Nrf2 from entering the nucleus and binding to the antioxidant response element (ARE). This process avoids the forming of heterodimers with small MAF proteins and activating the antioxidant genes. Maintaining the stability of this pathway relies on preventing Nrf2 from entering the nucleus and enabling its natural degradation within the cell [1–3, 91]

**Fig. 2:**
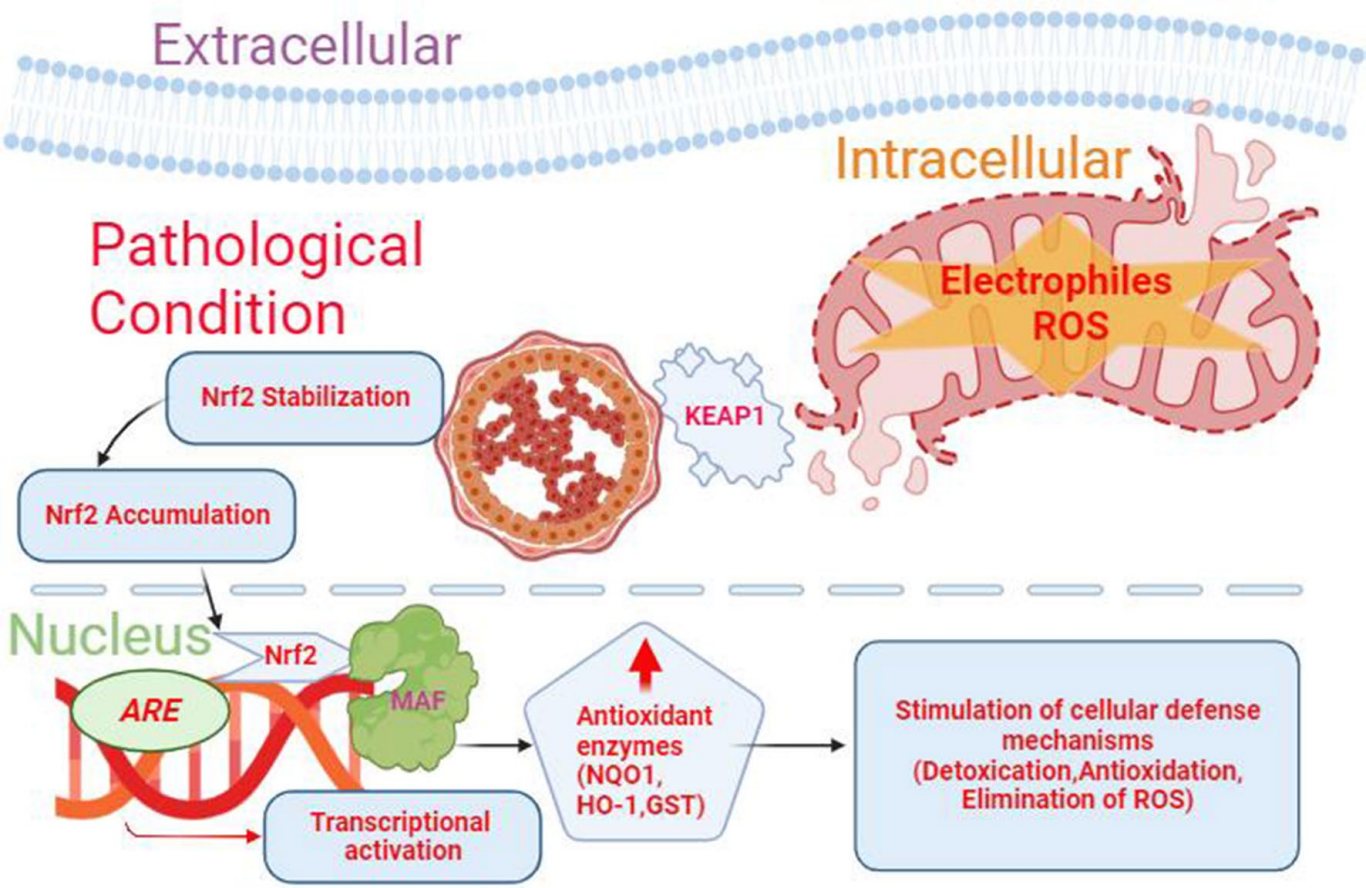
Schematic illustration of the initiation and roles of the Nrf2-NQO1 pathway in pathological conditions. During conditions of oxidative stress, reactive oxygen/nitrogen species (ROS) react with Kelch-like ECH-associated protein1 (KEAP1), leading to a conformational change that accumulates and releases nuclear factor erythroid 2-related factor 2 (Nrf2), preventing its degradation. Furthermore, small molecules can directly inhibit the Nrf2-KEAP1 interaction, resulting in the liberation of Nrf2. Consequently, Nrf2 translocates into the nucleus and forms heterodimers with small Maf proteins, binding to antioxidant response element enhancer (ARE) sequence and initiating the transcription of antioxidant enzymes, such as NAD(P)H Quinone Dehydrogenase 1 (NQO1), heme oxygenase-1 (HO-1), and glutathione S-transferase (GST). This triggers the activation of diverse cellular defense mechanisms and initiates the activation of numerous anti-oxidative stress genes. This coordinated response serves as a protective mechanism against cell damage and could be used as a therapeutic approach for various pathologies, including those affecting the central nervous system [1–3, 92]

The KEAP1-Nrf2 interaction plays a key role in preserving cellular balance by detecting oxidative stress and activating the necessary defense mechanisms. Under the physiological state, Nrf2 is subject to proteasome-dependent degradation by KEAP1. Nrf2 undergoes constant ubiquitination by KEAP1, leading to its degradation in the proteasome. Under non-stress conditions, this process promotes the ongoing degradation of Nrf2 protein within the cell (Fig. 1). The interaction between KEAP1 and Nrf2 plays a role as a sensor for oxidative stress. After exposure to oxidative stress (i.e., following hypoxia or inflammation), KEAP1 undergoes a conformational alteration, which prevents it from binding to Nrf2 and leads to the release, accumulation, and translocation of Nrf2 into the nucleus [1, 91] (Fig. 2). Nrf2 interacts with ARE in the promoter area of target genes in the nucleus [83]. Nrf2-ARE binding upregulates the expression of various genes by the enhancement of both transcription and translation. In the case of NQO1, Nrf2 binding to the ARE sequence located in the promoter region of the NQO1 gene leads to its increased expression [90, 92]. After NQO1 transcription, it is translated into an enzyme that helps to prevent the generation of ROS. Through its regulatory function, Nrf2 plays an essential role in enhancing the expression of NQO1 and other genes involved in the cellular defense pathway against the detrimental effects of oxidative stress (Fig. 2) [1, 92].

Furthermore, AMP-activated protein kinase (AMPK) has been revealed to activate the Nrf2-ARE-NQO1 signaling axis. Activation of AMPK leads to the phosphorylation of Nrf2 and promotes its nuclear translocation and subsequently leads to the activation of ARE-driven genes, including NQO1 [93]. This procedure enhances antioxidant enzyme levels and protects cells from oxidative stress [94]. Besides, the activation of AMPK-NQO1 signaling has been reported to modulate cellular metabolism, boost mitochondrial function, and augment ATP production, which may also contribute to neuroprotection [95]. NQO1 also plays a crucial role in regulating inflammatory responses via its modulation of AMPK and downstream signaling pathways [7]. Using in vitro cell line experiments, it has been proposed that the activation of Nrf2‑KEAP1‑antioxidant response element‑NQO1 signaling pathway could inhibit apoptosis and protect tissues from hypoxic insults [96]. The therapeutic potential of modulating this pathway might be a promising option for future studies.

### CREB-Nrf2/HO-1/NQO1 signaling pathway

The activation of the cAMP response element-binding protein (CREB) increases the expression of Nrf2 [97], which then enhances the expression of NQO1 via the upregulation of ARE. Moreover, the activation of the CREB signaling increases the expression of hypoxia-inducible factor 1-alpha (HIF-1α), an oxygen-dependent transcriptional activator. Consequently, HIF-1α has been shown to upregulate NQO1 expression in response to stressful events, such as hypoxia. Activation of CREB enhances the transcriptional activation of HO-1 in neurons in response to tissue injuries-induced oxidative stress, such as malignancy or hypoxia [98]. This pathway may represent a novel target through which the inflammatory disorders can be therapeutically conditioned [98]. There are bidirectional interactions between NQO1 and HO-1, where the activation of one protein can modulate the expression of the other. NQO1 promotes HO-1 expression through the activation of Nrf2 and upregulation of HO-1, in turn, can further enhance NQO1 expression [99].

### NQO1-Nrf2-PI3K/Akt pathway

The activation of the phosphoinositide 3-kinase (PI3K)/Akt signaling has been shown to increase the expression of NQO1 in several cell types, including pheochromocytoma cells and cancer cells [100, 101]. The PI3K/Akt pathway activation plays an important role in enhancing cell survival and growth through the phosphorylation of a variety of downstream targets involved in cell proliferation, metabolism, and apoptosis [102–105]. The activation of the PI3K/Akt signaling also promotes the detachment of Nrf2 from KEAP1, facilitates its translocation to the nucleus, and regulates Nrf2 activation in a ROS-dependent manner [106, 107]. The induction of the PI3K/Akt/Nrf2 signaling axis ameliorates oxidative stress and apoptosis via the regulation of Nrf2, NQO1, and HO-1 [108].

**Fig. 3:**
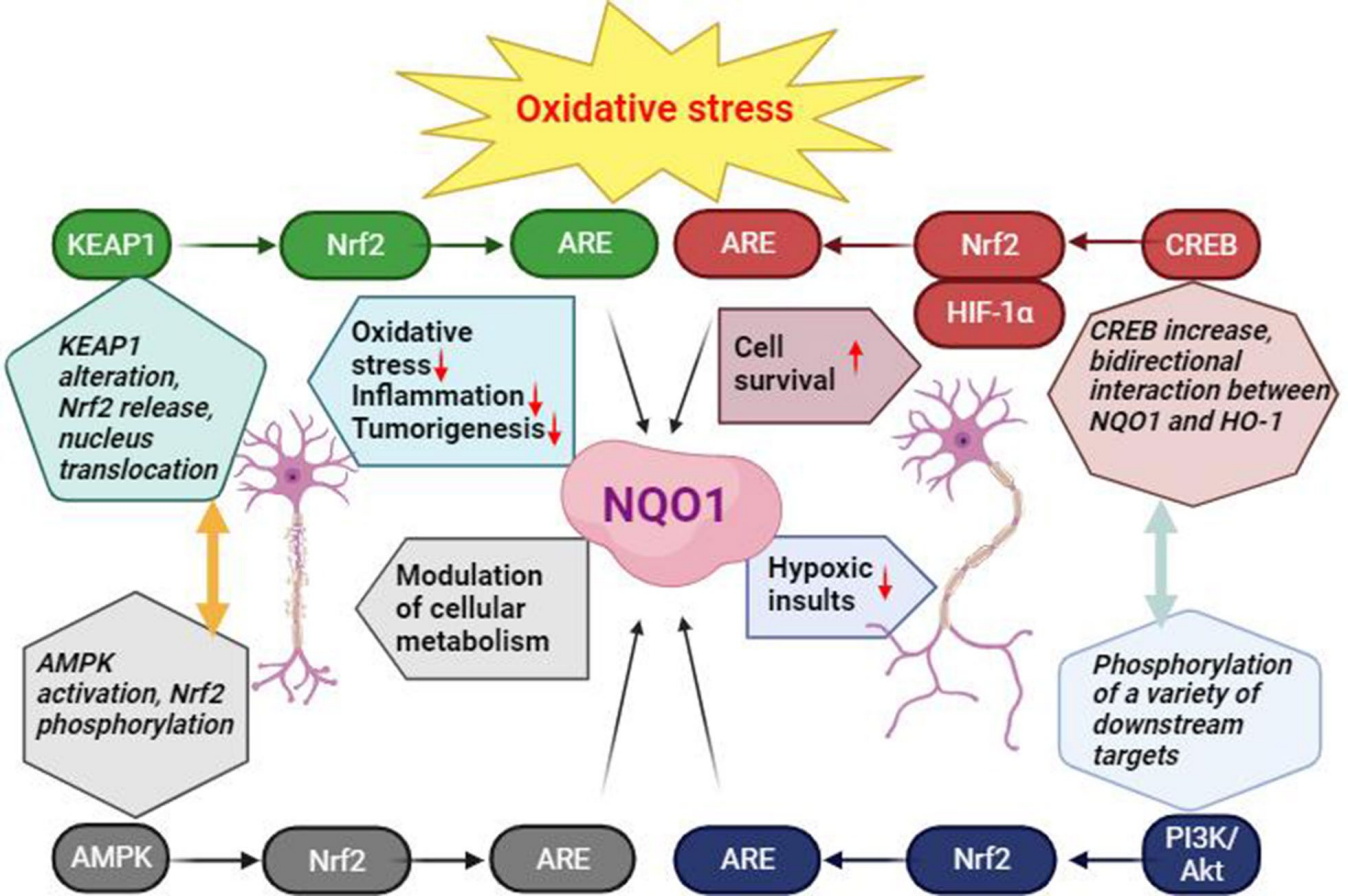
The interaction between the Nrf2-KEAP1-ARE-NQO1, CREB-Nrf2/HO-1/NQO1, and NQO1-Nrf2-PI3K/Akt signaling pathways plays a crucial role in the regulation of oxidative stress. The interplay between these pathways leads to a coordinated cellular defense against oxidative stress. Following the occurrence of oxidative stress, nuclear factor erythroid 2-related factor 2 (Nrf2) is a key player in the transcriptional regulation of various genes involved in the antioxidant response, including NQO1. The CREB-Nrf2/HO-1/NQO1 pathway can synergistically enhance the expression of antioxidant enzymes when Nrf2 accumulates. Moreover, NQO1 has a bidirectional modulator effect with the PI3K/Akt signaling pathway to further enhancement of the anti-oxidative response. The modulation of the interaction between these pathways on cellular metabolism and their role in addressing oxidative stress could be a potential shared therapeutic target for the treatment of various neurological disorders. NQO1 can well connect various pathways and act as a bridge. It mainly protects nerve cells by resisting oxidative stress, enhancing cell metabolism, promoting cell survival, and preventing hypoxia. Interactions between these pathways may vary in different cell types and under different conditions [84, 97, 109–112]

The interaction between the CREB-Nrf2 /HO-1/NQO1, Nrf2-KEAP1-ARE-NQO1, and NQO1-Nrf2-PI3K/Akt pathways implicates different and complex cellular processes linked directly and indirectly to the oxidative stress response. These pathways are interconnected, but their specific interactions in both physiological and pathological conditions depend on the specific cellular microenvironmental conditions [109, 110]. The regulatory effects of the interaction between these pathways on cellular metabolism and their role in addressing oxidative stress could be a potential shared therapeutic target for the treatment of various disorders, such as cancer, autoimmune disorders, and neurodegenerative diseases (Fig. 3) [111, 112].

## The involvement of NQO1 in neurological disorders

**Fig. 4:**
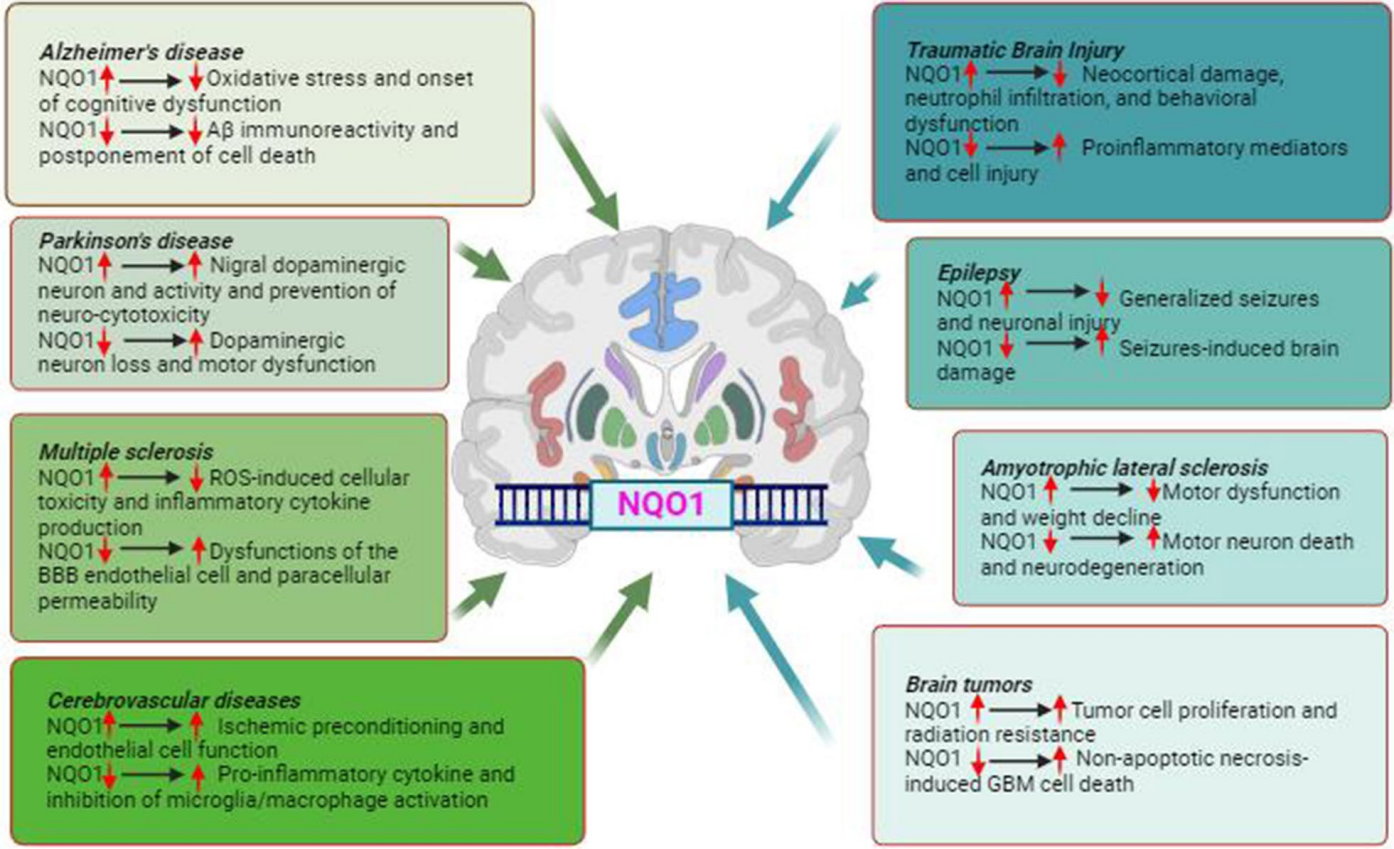
An overview of the potential effects of NQO1 alterations on the pathophysiology of various neurological disorders. Specific impacts of increased and decreased NQO1 values in each disease are outlined. The changes of NQO1 expression and function may contribute to the pathophysiology of various neurological disorders by affecting the neural tissues to counteract oxidative stress and maintain redox homeostasis. This could potentially lead to the alterations in susceptibility to neurodegeneration and neuroinflammation. The functions of NQO1 changes appear diverse and sometimes conflicting [113–116]

NQO1 is expressed in various cells within the CNS [113]. NQO1 is predominately expressed in astrocytes and a subgroup of oligodendrocytes, but lower levels of NQO1 are also detected in neuronal cell bodies, dendrites, and synapses [113, 114]. Numerous investigations have evaluated the potential role of NQO1 in the development and progression of various neurological disorders. These experimental and clinical assessments indicate that NQO1 may be implicated in the pathophysiology of a wide range of both acute and chronic neurological disorders, including epilepsy, AD, PD, amyotrophic lateral sclerosis (ALS), MS, cerebrovascular diseases, and brain malignant tumors (Table 1) (Fig. 4) [115].

**Table 1:** A concise overview of the role of NQO1 in diverse neurological disorders

CNS Disorders	The roles of NQO1 in CNS disorders				Experimental conditions	Methodologies	References
AD	Protective role against oxidative stress and inflammation	Regulation of genes involved in apoptosis	Aggregation kinetics of Aβ 1–42	Promotion of amyloid aggregation	Experimental studies (Human, rats, mouse tissues) Cell culture studies (B16,SH-SY5Y cells, glial cells, pyramidal cells)	Molecular biology, Histopathology, Immunohistochemistry, Behavioral assessments, Biochemical analysis	[72, 116–130]
PD	Protective effect on dopaminergic neurons	Prevent cell death, and modulate neurotrophin receptor affinity in PD	Impaired function of DJ-1 can lead to impairment of NQO1 function	Protect against neurotoxicity induced by MPTP	Experimental studies (Human, mouse tissues) Cell culture studies (Dopamine cells, CATH, SK-N-BE(2)C cells)	Molecular biology, Histopathology, Immunohistochemistry	[41, 131–156]
MS	NQO1 expression is upregulated in active and chronic active MS lesions	NQO1 and Nrf2 levels may be useful as biomarkers	Nrf2 inducers TFM-735, dimethyl fumarate, sulforaphane, and myricetin	Upregulation of NQO1 effects of dimethyl fumarate in the EAE model of MS	Experimental studies (Human, mouse tissues) Cell culture studies (Human primary cells, human brain endothelial cells) Clinical studies (Dimethyl fumarate, Natalizumab)	Molecular biology, Histopathology, Immunohistochemistry, Behavioral assessments	[10, 26, 157–173]
Cerebrovascular disease	Reduces oxidative stress after cerebral ischemia and subarachnoid hemorrhage	Upregulation of NQO1 plays a role in facilitating ischemic preconditioning	Nrf2-ARE pathway is activated after subarachnoid hemorrhage (SAH)	Nrf2-ARE-NQO1 pathway contributes to survival of astrocytes and neurons	Experimental studies (Human, rats, mouse tissues) Cell culture studies (Mouse brain microvascular endothelial cells) Clinical studies (Dimethyl fumarate)	Molecular biology, Histopathology, Immunohistochemistry, Behavioral assessments	[95, 174–201]
Traumatic Brain Injury	Contribution of oxidative stressgene expression process	Decreased neocortex injury, neutrophil infiltration, and microglia activation	Promoted the nuclear translocation of Nrf2	As a promising therapeutic strategy	Experimental studies (Human, rats, mouse tissues) Cell culture studies (Mouse brain microvascular endothelial cells)	Molecular biology, Histopathology, Immunohistochemistry, Enzyme assay, Neurobehavioral evaluation	[202–218]
Epilepsy	Upregulated in hippocampal tissue from individuals with epilepsy	Mitigating epilepsy and associated comorbidities	Reduces apoptosis and pro-inflammatory cytokines in epilepsy model	Protecting against seizures and epilepsy-induced brain damage	Experimental studies (Human, rats, mouse tissues) Cell culture studies (Sprague–Dawley rat mixed cortical neurons and glial cells)	Electrophysiological recordings, Molecular biology, Electron microscopy, Histopathology, Immunohistochemistry, Behavioral evaluation	[89, 113, 219–231]
ALS	Protect cells from oxidative stress by detoxifying ROS	Provides neuroprotective effects against neuronal degeneration	Activation of the Nrf2/ARE pathway	Reduce neuronal toxicity	Experimental studies (Human, mouse tissues) Cell culture studies (NSC-34,SOD1-G93A cells) Clinical studies (Fasudil, ferulic acid, caffeic acid,)	Molecular biology, Histopathology, Immunohistochemistry, Behavioral evaluation, Biochemical analysis	[232–247]
Brain tumors	Anti-oxidative stress response	Regulating the levels of ROS in glioblastoma	Impact on glioma therapy	Dual role in glioblastoma development	Experimental studies (Human, mouse tissues) Cell culture studies (SH-SY5Y cells, U87MG, LN229 GBM cells,T98G, LN-229,A172,U251 glioma cells) Clinical studies (Prognostic biomarker)	Molecular biology, Immunohistochemistry, Biochemical analysis, Bioinformatic analysis, Enzyme assay	[3, 44, 50, 59, 72, 248–268]

NQO1 plays an important role in diverse neurological disorders by the modulation of oxidative stress and cellular redox balance. Its upregulation or downregulation affects the pathophysiology of these neurological disorders. The table describes the complex roles of NQO1 in central nervous system disorders and highlights various experimental conditions and methodologies used by different preclinical and clinical studies. It provides an overview of NQO1's involvement in different neurological conditions, elucidating its impact within experimental settings and clinical investigations. ALS; amyotrophic lateral sclerosis, AD; alzheimer’s disease, Aβ; amyloid-β, CNS; central nervous system, MS; multiple sclerosis, NQO1; NAD(P)H Quinone Dehydrogenase 1, PD; Parkinson’s disease, ROS; oxidative stresses

### NQO1 and AD

Numerous pre-clinical and clinical studies indicate that NQO1 may play an important role in the evolution of AD. These investigations suggest that targeting NQO1 represents a potentially effective method for the treatment of AD. As previously discussed, NQO1 plays a neuroprotective role against oxidative stress and inflammation in the brain, which are well-known processes in the pathophysiology of AD. In an experimental AD mice model, the expression of NQO1 significantly increased in the hippocampus and neocortex in the initial stages of AD, which was followed by a progressive decline during the further development of AD-like pathology in comparison to wild-type mice [116]. Long-term administration of Nrf2 inducer enhances NQO1 expression and prevents the onset of cognitive dysfunction by inhibiting oxidative stress and neuroinflammation in a knockdown mouse model of AD [117]. NQO1 has been found to posttranscriptionally regulate numerous genes involved in apoptosis, and massive neuronal death due to apoptosis is a well-known characteristic of AD pathophysiology [118]. The presence of C609T polymorphism resulted in significantly faster aggregation kinetics of amyloid-β (Aβ)1–42 than with wild-type NQO1. Moreover, the application of dicoumarol, an NQO1 inhibitor that binds in close proximity to the FAD-binding site of the enzyme and stabilizes it, significantly reduced the aggregation kinetics of Aβ1-42 [119]. Using murine melanoma cell line B16, depletion of riboflavin has been shown to induce instability of NQO1 and promote amyloid aggregation in cells [120]. The application of NXPZ-2, a small molecule that disrupts the interaction between KEAP1 and Nrf2 proteins, in an Aβ1-42 oligomer-injected mouse model led to an upregulation of Nrf2 and NQO1 in the whole cell lysate and enhanced nuclear translocation. This treatment led to decreased Tau values in the hippocampus and neocortex of the AD mice [121]. Treatment with deferiprone (an oral iron chelator) rescued the expression of NQO1, restored memory, and increased hippocampal brain-derived neurotrophic factor (BDNF) levels in an experimental animal model of memory impairment [122]. DHPA, an antioxidant and anti-inflammatory substance, inhibited Aβ1-42/Cu^2+^/ascorbic acid-induced oxidative damages by regulating mitochondrial apoptosis in human neuroblastoma SH-SY5Y cells, inducing the KEAP1/Nrf2/HO-1 signaling axis and enhancing the expression of NQO1 [123].

Numerous studies indicate that neurons in the substantia nigra (SN) experience persistent oxidative stress. This is supported by observations that both individuals with AD and controls have exhibited greater neuronal NQO1 activities in this particular area of the brain [124]. Investigations assessed brain tissues from patients with AD have reported varying results regarding NQO1 alterations. Postmortem studies on hippocampal tissues from patients with AD have shown an enhancement of NQO1 expression in their neurons compared to young and age-matched control autopsies. This investigation has identified two different forms of NQO1 expressions; *i)* NQO1 was detected within intracellular neurofibrillary tangles, neuropil threads, and pre-neurofibrillary tangles, and *ii)* NQO1 was identified as cytoplasmic staining in hippocampal pyramidal neurons [125]. Some studies found NQO1 upregulation in glial cells and later in hippocampal neurons in AD patients, suggesting that increases in NQO1 enzymatic activity may exert a neuroprotective effect [126]. The upregulation of NQO1 in hippocampal pyramidal cells of subjects with AD suggested that NQO1 may be involved in the brain neuroprotective mechanism that is activated in response to the pathological processes of AD [127]. However, another investigation reported that approximately half of patients with AD may lack NQO1 expression in the hippocampus due to a C609T polymorphism [72, 118]. It has been suggested that the age-dependent accumulation of NQO1 may be impaired in individuals with AD. Evaluation of hippocampi from patients with AD revealed that the NQO1 protein level was undetectable in 50% of the cases due to the presence of the C609T polymorphism [127]. AD is a heterogeneous disease and this discrepancy between these studies could be attributed to the broad spectrum of genotype and phenotype as well as various interactions with environmental factors in AD [128]. Moreover, evidence suggests that the NQO1 gene polymorphism C609T may not be an additional genetic risk factor for late-onset AD [129]. Targeting Nrf2 and its downstream genes, including NQO1, could provide potential benefits for the management of AD through the activation of antioxidant genes, the suppression of microglia-mediated inflammation, and the promotion of mitochondrial function [130].

### NQO1 and PD

PD, a progressive neurodegenerative condition, is identified by the gradual degeneration of dopaminergic neurons and loss of dopamine (DA) production in the SN [131]. A growing body of evidence suggests that oxidative stress and redox dysfunction play a crucial role in the vulnerability of DA cells to harmful events within the brain and may be involved in the pathophysiology of PD [132]. Several studies have suggested that NQO1 exerts a protective effect on DA cells, presumably by alleviating oxidative stress-induced damages [41, 133, 134]. BDNF may contribute to the pathogenesis of PD by the activation of the Nrf2-ARE-NQO1 signaling pathway, which promotes interactions between astrocytes and neurons, protects dopaminergic neurons from oxidative damage, prevents ferroptosis-induced cell death, and modulates the affinity of neurotrophin receptor [135, 136]. Appropriate activation of NQO1 is dependent on the proper function of DJ-1, a multifunctional protein that regulates cellular metabolism [137]. Loss of DJ-1 was accompanied by a decrease in the stability of Nrf2 pathway activity and deficits in NQO1 expression [138]. NQO1 is expressed in the SN and has been found to be markedly increased in PD patients [139]. The expression values of NQO1 are significantly enhanced in the early and intermediate stages of PD, but decrease in the end-stage of the disease [139]. It has been shown that Akt phosphorylates NQO1, which promotes its ubiquitination and subsequent degradation. Parkin, an E3 ubiquitin ligase, is involved in a procedure that ultimately impacts the antioxidant efficacy of NQO1 in an animal model of PD [140]. Analysis of postmortem specimens from subjects with PD revealed that cytoprotective proteins associated with Nrf2 expressions, such as NQO1 and p62, were partially sequestered within Lewy bodies. This observation suggests a diminished neuroprotective capacity of the Nrf2 signaling pathway in PD [141]. Besides, chlorhexidine, an antimicrobial substance, has been shown to provide cytoprotection in a neurotoxic cell model of PD by disrupting KEAP1-Nrf2, which leads to the nuclear translocation of Nrf2 and subsequent upregulation of downstream genes, including NQO1 [142]. A meta-analysis evaluated the association of NQO1 and tumor necrosis factor (TNF) polymorphisms with PD in data obtained from 15 studies comprising 2,858 patients with PD and 2,907 healthy controls. The findings indicated that TNF-1031 polymorphism may pose a risk for PD under either recessive or additive models. However, the meta-analyses did not support the involvement of NQO1 C609T and TNF-308 in PD risk [143].

MPTP, a neurotoxin, is commonly used to induce PD-like features in rodents [144–146]. The application of MPTP augmented hydroxyl radicals and oxidative stress and enhanced intracellular ROS in the rat SN, which ultimately led to cell death [147, 148]. The activation of NQO1 through the use of sulforaphane, a natural compound with antioxidant and anti-inflammatory properties, can provide protection against MPTP-induced neurocytotoxicity in vivo [149] as well as against 6-hydroxydopamine-induced cell injury in vitro [150]. Several other compounds, such as vinyl sulfoxide and isothiocyanate compound 3, activate the Nrf2-KEAP1 signaling pathway and increase mRNA and protein levels of the Nrf2-dependent antioxidant enzymes, including NQO1, in lipopolysaccharide-activated BV-2 microglial cells [151, 152]. Treatment with the indole derivative NC009-1 improved both motor impairment and depressive behaviors and also increased DA and DA transporter values in the SN. Furthermore, administration of NC009-1 reduced oxidative stress and decreased microglia and astrocyte activity in the ventral midbrain of mice after exposure to MPTP. These beneficial effects were accompanied by an upregulation of Nrf2 and NQO1 as well as a downregulation of iNOS, TNF-α, IL-6, and IL-1β [153]. Moreover, isoliquiritigenin, a flavonoid compound, has been shown to improve motor deficits in a PD mice model through the upregulation of Nrf2 and NQO1, while concurrently downregulating proinflammatory mediators [154]. The application of dimethyl fumarate increased Nrf2 and NQO1 values in the basal ganglia and simultaneously protected dopaminergic neurons from α-SYN-induced toxicity and alleviated microgliosis and astrocytosis in the mouse SN [141]. Although studies have shown that susceptible variants of NQO1 genes could interact with some pesticides and increase PD risk [155], a meta-analysis evaluating sixty-four studies evaluating the relationship between pesticide exposure and PD revealed no significant association between exposure to pesticides and alteration of NQO1 [156]. Further investigation into the modulation of NQO1 function without causing adverse effects and evaluation of additional molecules that interact with NQO1 in the context of PD, particularly on post-mortem specimens or induced pluripotent stem cell-derived neuronal cultures from PD patients, could potentially reveal new therapeutic opportunities.

### NQO1 and MS

Oxidative stress is implicated in the onset and progression of MS [157]. NQO1 expression in the CNS of patients with MS may serve as a biomarker of oxidative stress. Both clinical and experimental studies suggest that NQO1 upregulation may indicate an inherent protective response against ROS-induced toxicity in MS [26]. The protein values of NQO1 and Nrf2 were markedly reduced in human brain microvascular endothelial cells following exposure to IgG purified from sera of individuals suffering from myelin oligodendrocyte glycoprotein antibody-associated disorder [158]. The concurrent presence of inflammation and astrocytic NQO1 expression has been observed in patients with MS. Assessment of brain tissues obtained from ten autopsy MS patients as well as seven age and region-matched controls revealed that NQO1 expression was primarily identified in astrocytes located at the center and rim of the chronic active plaque. A direct association was observed between the number of NQO1-expressing astrocytes and the infiltration of T-cells. Moreover, a significant correlation was reported between the mean number of NQO1-expressing astrocytes and astrocytes expressing peroxiredoxins [159]. Evaluation of 64 brain lesions from 14 patients with clinically diagnosed and neuropathologically confirmed MS revealed increased NQO1 expression in macrophages and astrocytes within active and chronically active demyelinated lesions. It has been suggested that NQO1 upregulation in these lesions could be induced by ROS derived from macrophages and serve as a protective mechanism against ROS-induced cellular toxicity [26]. Genetic nuances associated with NQO1could impact could impact the activity or expression of this enzyme and may have implications for an individual's susceptibility to MS. In a case–control investigation involving 231 MS patients and 380 controls, an association between the C609T inborn polymorphism of NQO1 and the risk of developing primary progressive MS has been reported in the Greek population [157]. However, it should be noted that another study conducted on the Spanish Caucasian population did not validate this association [160].

Several studies have demonstrated that the induction of the Nrf2 signaling pathway can suppress the development and progression of disease in various animal MS models [161]. Using human primary cell profiling systems in the experimental autoimmune encephalomyelitis mouse model (EAE) of MS, TFM-735, a potent Nrf2 inducer, has been shown to enhance NQO1 levels in the brain and spleen, inhibit inflammatory cytokine production, and ultimately prevent disease progression [162]. Furthermore, the application of dimethyl fumarate, an antioxidant medicament and Nrf2 inducer approved for the treatment of MS, resulted in a higher expression of the NQO1 in various in vitro and in vivo experimental models [163, 164]. The upregulation of NQO1 is an essential part of the anti-oxidative/inflammation cascade that contributes to the effects of dimethyl fumarate in the EAE model [165]. Furthermore, greater protection of human astrocytes following the application of dimethyl fumarate is associated with the activation of NQO1 [165]. In an in vivo mouse model of MS, sulforaphane, an organosulfur compound, and Nrf2 inducer, has been shown to decrease oxidative stress levels in the brain. This effect is linked to enhanced activation of the Nrf2/ARE pathway, which leads to increased levels of the antioxidant enzymes HO-1 and NQO1 [166]. Furthermore, melatonin has been shown to suppress lymphocytic infiltration and oxidative stress levels and was associated with a decrease in disease progression in the EAE mouse model. This effect is also thought to be due to the activation of the Nrf2/ARE pathway and the subsequent increase in values of the antioxidant enzymes HO-1 and NQO1 [167]. Myricetin, a flavonoid commonly found in various plants, has been shown to reduce demyelinating lesions by promoting Nrf2 translocation to the nucleus and enhancing the expression of HO-1 and NQO1 in a cuprizone-induced demyelination model of MS [168].

An assessment of the impact of dimethyl fumarate on peripheral immune cells in patients with MS indicated that subjects with higher values of NQO1 after receiving dimethyl fumarate were more likely to have no evidence of disease one year later. Furthermore, the extent of NQO1 induction has shown a correlation with the age of the patient [169]. A statistically significant increase in NQO1 expression was observed in MS patients treated with dimethyl fumarate compared to their baseline levels and placebo-treated patients [170]. A significantly greater frequency of both homozygous and heterozygous NQO1 C609T genotype has been reported among patients suffering from MS compared to controls. MS patients have shown a 1.5-fold enhanced risk of carrying at least one variant of the NQO1 C609T genotype in comparison to healthy individuals [157]. A case–control study involving 254 MS patients and 370 healthy donors has revealed a significantly greater occurrence of NQO1 variant genotypes in patients with relapsing–remitting MS compared with controls, suggesting the role of the NQO1 gene as a risk factor for MS [10]. Evaluation of 130 patients with MS has shown a significantly enhanced frequency of NQO1 mutant polymorphisms documented in non-responder MS patients following natalizumab administration compared to the responders. Polymorphisms in the NQO1 gene may be a key factor in the treatment of MS patients in the future. Patients who carry the wild-type genotype or only one polymorphism have shown a better clinical outcome following the treatment with natalizumab, a humanized monoclonal antibody used to treat MS [171]. The application of the primary metabolite of dimethyl fumarate, monomethyl fumarate, which is currently used in the clinics for the treatment of relapsing–remitting MS, significantly induced NQO1 in inflamed human brain endothelial cells and decreased monocyte transendothelial migration. These findings suggest beneficial therapeutic effects on the inflamed blood–brain barrier in MS patients [172]. Both dimethyl fumarate and monomethyl fumarate induced NQO1 gene expression in ex vivo-stimulated human peripheral blood mononuclear cells obtained from 200 MS patients treated with dimethyl fumarate [170]. In addition to its role as a therapeutic target, the Nrf2-NQO1 pathway has been proposed as a potential biomarker for predicting and evaluating treatment outcomes in MS [169, 173].

### NQO1 and cerebrovascular diseases

Growing evidence suggests a potential association between NQO1 and the pathogenesis of various cerebrovascular diseases, including ischemic stroke and cerebral infarction. The expression of NQO1 is upregulated in response to hypoxia/reoxygenation insults, and this upregulation plays a role in facilitating ischemic preconditioning through the reoxygenation-dependent Nrf2 pathway [174]. The Nrf2-ARE-NQO1 signaling pathway is not only involved in maintaining cerebral blood flow but could contribute to the survival of astrocytes and neurons following cerebral ischemia–reperfusion injury [175]. Several studies have shown that NQO1 expression and activity are altered in animal models of stroke as well as in patients with stroke. A study on 141 patients with ischemic stroke and 139 matched control subjects has shown a significant association between NQO1 gene polymorphisms and the incidence of ischemic stroke. This investigation revealed a correlation between the cc genotype of NQO1 rs1800566 and the occurrence of ischemic stroke [176]. An investigation on the clinical efficacy of butylphthalide in 127 patients with acute ischemic stroke revealed a link between the improvement of the cerebrovascular vascular reserve function and enhancement of the establishment of collateral compensatory vessels with changes in the expression of Nrf2‑KEAP1‑ARE‑NQO1 signaling pathway. Following the administration of butylphthalide in patients with stroke, the serum levels of Keap1 were elevated, while the serum levels of NQO1, Nrf2, and ARE were reduced compared to those in the control group [177]. Simultaneously conducting clinical research (with 100 patients) and an animal study revealed that dimethyl fumarate exerted a neuroprotective effect against post-stroke cognitive impairment through its antioxidative properties. Moreover, dimethyl fumarate improved cognitive impairment induced by ischemic stroke. This study also showed higher expression values of NQO1 in the rat ischemic brain that received dimethyl fumarate in comparison to the Sham group [178]. Furthermore, a case–control study involving postmortem tissues of 26 patients with stroke due to supratentorial intracerebral hemorrhage, it has been shown that NQO1 expression was significantly higher in the peri-hematoma area compared to distant ipsilateral brain tissue obtained 7 days after the onset of hemorrhage [179]. The rats treated with dimethyl fumarate and subjected to a middle cerebral artery occlusion (MCAO) showed enhanced performance in Morris water maze and shuttle box tasks, as well as a more orderly arrangement of Nissl bodies and neurons, fewer apoptotic cells and autophagosomes, and higher NQO1 expression, compared to the control rats [178]. After MCAO, dimethyl fumarate acts as an effective immunomodulator by decreasing pro-inflammatory cytokines, inhibiting the infiltration of neutrophils and T-cells, and reducing the number of activated microglia/macrophages in the brain infarct zone within 1–2 weeks [180]. In this rat model, the administration of dimethyl fumarate resulted in reduced neurological deficits and infarct size, which was accompanied by increased expression levels of Nrf2, HO-1, and NQO1 [181]. Rev-erbα, a circadian clock protein, plays a complex role in the regulation of inflammation and oxidative stress [182]. Rev-erbα ameliorates acute neurological deficits, reduces infarct volume, and enhances the activation of Nrf2 and its downstream targets HO-1 and NQO1 following an ischemic event in mice [183]. In male rats treated with MCAO and reperfusion, inhibiting Cullin 3, a member of the ubiquitin ligase family, suppressed the ubiquitin-mediated degradation of Nrf2, increased the nuclear translocation of Nrf2, and upregulated the expression of HO-1 and NQO1 [184]. Pharmacological and/or dietary induction of the Nrf2/HO-1/NQO1 pathway may serve as a therapeutic strategy to prevent oxidative stress-induced damage in the peripheral vessels or the blood–brain barrier in stroke [185]. Sulforaphane significantly activated Nrf2 and upregulated mRNA expression of HO-1 and NQO1 in mouse brain microvascular endothelial cells adapted to hyperoxic and normoxic O_2_ levels. Inhibition of Nrf2 transcription in these cells led to the loss of HO-1 and NQO1 and a significant increase in free radical generation upon reoxygenation [186]. The induction of the Nrf2-ARE-NQO1 pathway by sulforaphane additionally provides protection to developing neurons and astrocytes against cell death induced by ischemic injuries [187].

In a rat model of cerebral ischemia, the inhibition of mitochondrial dihydrolipoamide dehydrogenase, a potential target for protection against ischemic insults, resulted in the upregulation of NQO1 expression. This intervention led to a reduction in oxidative stress, a decrease in cell injury/death, and an increase in mitochondrial ATP output [188]. The application of an enriched micromilieu, an environment that is designed to provide increased cognitive, sensory, and motor stimulation, has been shown to be an effective approach for improving cognitive dysfunction in cerebrovascular diseases [189]. An enriched environment has been shown to enhance cognitive function in rats with post-stroke cognitive impairment through the suppression of neuroinflammation and oxidative stress. This effect was accompanied by the upregulation of mRNA expression of Nrf2 and its target genes, NQO1 and HO-1 [190]. The induction of the AMPK-Nrf2-NQO1 signaling pathway has been shown to protect hippocampal cells from oxygen–glucose deprivation-reoxygenation-induced injury [95]. Targeting chromobox 7, a transcriptional repressor, has been shown to influence Nrf2, HO-1, and NQO1 levels as well as behavioral and pathological outcomes in rat ischemic brains in vivo [191].

Experimental studies have also shown that the induction of the Nrf2-ARE pathway in the brain following subarachnoid hemorrhage (SAH) leads to an enhancement of Nrf2-ARE-related factors, including Nrf2, HO-1, and NQO1 [192]. Increased expression of Nrf2-ARE pathway-related agents, such as Nrf2 and NQO1, attenuated SAH-induced early brain injury in rats [193–195]. Geniposide, an anti-inflammatory and anti-oxidative herbal compound, has been shown to reduce brain edema, attenuate blood–brain barrier permeability, inhibit neurocyte apoptosis in the temporal cortex, and improve behavioral function following SAH in rats. These effects were associated with decreased levels of ROS and upregulation of Nrf2 and NQO1 levels [196]. The use of RTA 408, an Nrf2 inducer, can attenuate SAH-induced morphological damages by the enhancement of Nrf2, HO-1, and NQO1 values in a rodent model of SAH [197]. Another Nrf2 inducer, tert-butylhydroquinone, upregulated the expression of KEAP1, Nrf2, HO-1, and NQO1, and ameliorated brain edema, blood–brain barrier dysfunction, behavioral impairment, and cell injury/death after SAH in a rat in vivo model [198]. Propofol, a widely used intravenous anesthetic with sedative and hypnotic properties, has been shown to have neuroprotective effects after ischemic insults and traumatic brain injury (TBI) [199]. It has been demonstrated that propofol attenuates SAH early brain injury through the upregulation of the immunoreactivity of p-Akt, Nrf2, and NQO1 in adult rats in vivo [200]. The future of targeting NQO1 as a potential therapeutic intervention needs a deeper understanding of the complex mechanisms regulating the action of the Nrf2-NQO1 signaling pathway in the development of cerebrovascular diseases. With limited clinical data available, there is a need for additional clinical studies to assess the safety and effectiveness of targeting NQO1 for the treatment of cerebrovascular diseases [201]. Investigating the synergistic effects of combined therapies (e.g., NQO1 modulators in combination with traditional/conventional therapies) could provide insights into maximizing therapeutic outcomes.

### NQO1 and TBI

Oxidative stress plays a substantial role in mitochondrial dysfunction and activation of excitatory pathways in both acute and late phases of TBI [202]. The Nrf2 pathway exerts a neuroprotective role in TBI by the alleviation of oxidative damage and neuroinflammatory responses [203]. The fluid percussion injury model, a commonly used experimental approach, provides valuable insights into TBI research, particularly for clinical TBI without skull fracture [204]. The downregulation of Nrf2 and its downstream factor NQO1 in TBI leads to the activation of proinflammatory mediators, such as TGF-β1 and NF-kB, and exacerbates cell injury and death in a fluid percussion injury mouse model of TBI [205]. The genetic deletion of Nrf2 hampers the recovery of both motor and cognitive functions after TBI [206]. The human brain is a suitable tissue for TBI research if it possesses characteristics suitable for modeling TBI [207]. Investigations conducted on tissues obtained during neurosurgery from 31 patients with TBI have revealed that the activation of the Nrf2 pathway may provide neuroprotection against TBI secondary brain injury. These patients were adults with surgical indications, such as progressive cognitive deficits and neurological impairment with no prior neurological disease. Five other specimens of control brain tissue were obtained from patients with benign tumors. In these traumatic brain specimens, significant increases in the nuclear value and decreases in the cytoplasmic level of Nrf2 were associated with an enhancement of the NQO1 and GST upregulation [208].

Several substances targeting the Nrf2 pathway and its downstream factors exert an appropriate protective effect against the detrimental procedures of TBI in the brain [209]. Sulforaphane, an Nrf2 activator, induces NQO1 activity and improves the function of the neurovascular unit following TBI by reducing the loss of endothelial cells and tight junction proteins and preserving blood–brain barrier integrity [210]. The application of curcumin, an anti-oxidative natural substance, decreased neocortex injury, neutrophil infiltration, and microglia activation, induced the nuclear translocation of Nrf2, and enhanced the expression of downstream targets such as HO-1and NQO1 in mice following TBI [211]. Furthermore, the attenuation of neuroinflammatory response through the administration of dexmedetomidine has been shown to prevent neuronal apoptosis and improve behavioral function after TBI in rats. This was accompanied by the promotion of Toll-like receptor 4 expression and upregulation of HO-1 and NQO1 [212, 213]. The administration of tert-butylhydroquinone, an antioxidant compound, reduced the volume of the hypoxic lesions and improved motor function and cognitive deficits after TBI in a cortical impact model in rats. These beneficial outcomes were associated with a significant increase in the nuclear accumulation of Nrf-2 and expression of HO-1 and NQO1 [214]. Targeting mitochondrial oxidative stress by mitoquinone, a strong mitochondrial-targeted antioxidant, has shown promising results in improving neurological deficits, alleviating brain edema, and inhibiting neuronal apoptosis in a rodent model of TBI. This approach is associated with accelerated Nrf2 nuclear translocation and consequently the upregulation of HO-1 and NQO1 [215]. The administration of erythropoietin significantly enhanced the mRNA expression and activities of Nrf2 and its downstream enzyme NQO1, leading to a significant improvement in secondary TBI brain injury in mice [216]. Administration of Irisin, a hormone-like protein that is produced in response to physical activity, increased expression of NQO1 that may contribute to the neuroprotective effects associated with endurance exercise in TBI [217]. Comprehensive studies of how exercise interventions might be optimized post-TBI to harness NQO1-related neuroprotection have the potential to be groundbreaking. Exploring the modulation of the Nrf2 pathway and its downstream factors for alleviating the detrimental effects of TBI and promoting neuroprotection offers exciting prospects for future studies [209]. Furthermore, the integration of multi-omics data may provide a comprehensive understanding of TBI pathogenesis and recovery, with NQO1 as a potential focal point [218].

### NQO1 and epilepsy

Chronic inflammation induces mitochondrial dysfunction by generating ROS, which in turn leads to mutations in mitochondrial DNA. This process contributes to neuronal cell injury and loss in patients with epilepsy, especially individuals with medically intractable epilepsy [219, 220]. The Nrf2 signaling pathway has appeared as a critical mechanism for combating seizure-induced ROS, cell death, and persistent seizures in epilepsy. A notable upregulation of Nrf2 mRNA expression was detected in human brain tissue obtained from patients with epilepsy [221]. A study involving 31 temporal lobe specimens obtained during epilepsy surgery revealed a significant increase in Nrf2 mRNA expression compared to autoptic tissues. Furthermore, using an adeno-associated virus to overexpress human Nrf2, the expression levels of Nrf2 and NQO1 increased progressively in mice, reaching a peak at 72 h after pilocarpine-induced status epilepticus. The mice injected with the virus exhibited significantly fewer generalized seizures, reduced microglia activation, and less damage to hippocampal neurons [221]. NQO1 expression in the human epileptic brain may also be linked to the outcomes of epilepsy surgery. An investigation of the brain specimens from 26 patients who underwent surgery for medically refractory epilepsy revealed a significant reduction of NQO1 expression among patients who experienced aura after surgery (ILAE class 2), in comparison to seizure-free patients (ILAE class 1) [222]. Activation of this pathway has been suggested as a potential interventional approach to alleviating epilepsy and associated comorbidities [223]. To investigate the in vivo functions of NQO1, NQO1-/- mice have been generated [224]. It has been reported that some of the NQO1 (− / −) mice exhibit seizures, which are characterized by tail tension, bending of the front legs, rolling onto their side, salivation, and occasional spasms [113]. Interestingly, the administration of valproic acid, an antiepileptic drug, to the mouse embryo tissues after treatment with the Nrf2 antioxidant pathway inducer, 1,2-dithiole-3-thione, significantly increased the expression of NQO1 [225]. These findings indicate the implication of NQO1 in the modulation of brain excitability.

The expressions of nuclear Nrf2, HO-1, and NQO1 were upregulated 24 h after the onset of lithium chloride-pilocarpine-induced status epilepticus in sixty male Sprague–Dawley rats. However, the extent of this upregulation was insufficient to effectively counteract oxidative stress damage, attenuate lipid peroxidation, and reduce apoptosis [226]. Enhancement of nuclear Nrf2, NQO1, and HO-1 by the application of hydroxylated fullerenes, known as a potent free radical scavenger, significantly reduced lipid peroxidation and apoptosis in the hippocampus of rats following status epilepticus [226]. The administration of dimethyl fumarate, an activator of the Nrf2 pathway, significantly reduced seizures, suppressed pro-inflammatory cytokines, and enhanced the gene expression of Nrf2, NQO1, and HO-1 in the chemical kindling epilepsy rat model [227]. Comparable outcomes have been observed in fifty male Wistar rats subjected to amygdala kindling [89]. Nrf2 expression levels, along with the three Nrf2-regulated genes HO-1, NQO1, and GST, exhibited a notable increase in mice with status epilepticus. The overexpression of human Nrf2 using an adeno-associated virus vector led to a marked decrease in generalized seizures as well as a reduction in microglia activation and neuronal injury in these mice [228]. In a study on 80 male Sprague–Dawley rats, genistein, a naturally occurring isoflavone substance, counteracted the reduction of Nrf2, HO-1, and NQO1 levels following recurrent seizures induced by pentylenetetrazol. This effect is accompanied by a prolongation of seizure latency and a reduction in seizure intensity and duration of generalized seizures. Moreover, this substance exhibits a protective effect on epilepsy-induced brain damage [229]. A total of 60 adult male Wistar rats were used to evaluate the antiepileptic and neuroprotective effects of dehydroepiandrosterone, a steroid hormone, against iron-induced epilepsy [230]. The iron-induced epileptiform burst discharges in rats simulate post-traumatic epilepsy in humans [231]. Treatment with dehydroepiandrosterone, a steroid hormone, has also demonstrated the ability to suppress oxidative stress, reduce neuronal apoptosis, and improve electrophysiological alterations in an iron-induced epilepsy model in rats. This alleviative effect was associated with the upregulation of Nrf2 and NQO1 [230].

### NQO1 and ALS

The main pathological mechanisms of ALS include oxidative stress, neuroinflammation, and mitochondrial dysfunction [232, 233]. Several experimental in vitro and in vivo studies as well as clinical trials revealed that the KEAP1-Nrf2 pathway as well as its downstream mediators has been recognized as key players in the evolution of ALS. Perturbation in the Nrf2/ARE signaling pathway, which is characterized by alterations in Nrf2 expression, may contribute to the chronic degeneration of motor neurons in ALS [234]. In postmortem brain and lumbar spinal cord tissues obtained from 5 patients with ALS, a reduction in both Nrf2 mRNA and protein expression levels has been observed in neurons within the motor cortex and spinal cord compared to autoptic specimens from 5 individuals without any neurological or psychiatric disease, suggesting an impaired capacity of these cells to counteract oxidative stress. These alterations were associated with an enhancement of KEAP1 mRNA expression, particularly in the motor cortex [235]. The activation of the Nrf2/ARE pathway in a transgenic familial ALS mice model demonstrated significant clinical benefits, including reduced weight decline, improved motor impairments, and prolonged survival. Furthermore, this effect was assessed in NSC-34 SOD1-G93A cells, a specialized cell line derived from motor neuron-like cells that have been transfected to express a mutant form of the human superoxide dismutase 1 (SOD1) gene. The activation of the Nrf2 pathway in these cells resulted in a significant upregulation of Nrf2-regulated genes, including NQO1, GST, and HO-1 [236]. Furthermore, in vitro exposure of astrocytes isolated from the spinal cord of symptomatic SOD1-G93A mice and human astrocytes to mesenchymal stem cell-derived extracellular vesicles reduced their neurotoxicity towards motor neurons, potentially mediated by miRNAs carried by the extracellular vesicles. Transfecting human astrocytes with miR-29b-3p leads to the upregulation of NQO1 antioxidant activity and a reduction in neurotoxicity towards motor neurons. This study suggests the therapeutic potential of extracellular vesicles in various subtypes of ALS [237]. It has been reported that familial forms of ALS are accompanied by mutations in the SOD1 gene. The elimination of the Nrf2 gene in SOD1-G93A mice exerted a significant effect on NQO1 expression among the other recognized Nrf2-regulated enzymes. Knocking out Nrf2 in these transgenic mice resulted in accelerated motor neuron loss and astrocytic proliferation, and led to earlier disease onset and shorter lifespan [238]. Investigation on the lymphoblasts of 10 patients (six sporadic ALS and four male SOD1-ALS patients) has shown an increase in Nrf2, HO-1, and NQO1 protein values in patients with sporadic ALS. However, in lymphoblasts carrying mutations in the SOD1 gene, only a significant decrease in the NQO1 mRNA levels has been observed. This suggests a potential disruption in the transcriptional regulation of NQO1 in the presence of mutated SOD1 in ALS [239]. An investigation on lymphoblasts derived from 10 patients with ALS (six sporadic ALS and four SOD1-ALS patients) revealed that the baseline levels of the Nrf2 in lymphoblasts derived from patients with SOD1-related ALS were comparable to those of control cells derived from seven healthy subjects. Treatment with combined structural fragments fasudil and the Nrf2 inductors/radical scavengers ferulic and caffeic acids led to a significant increase in NQO1 levels at both the mRNA and protein values [240]. These findings point to the potential therapeutic efficacy of targeting NQO1 in ALS subjects with mutations in the SOD1 gene.

The implication of ATP-gated P2X7 ion channel in the progression of ALS has been identified [241]. In a study involving 47 SOD1-G93A mice, the inhibition of the ATP-gated P2X7 ion channel by the CNS-penetrant P2X7 antagonist JNJ-47965567 resulted in a significant reduction in NQO1 gene expression [242]. A cell culture study revealed that mutations in TANK-binding kinase 1 and sequestosome 1, which are genetic risk factors for ALS, disrupted selective autophagy in association with a dysregulation in the levels of NQO1 and KEAP1 [243]. Pathological transactive response DNA-binding protein 43 (TDP-43) is also involved in the pathogenesis of ALS [244]. In an ALS cell model featuring human mutant TDP-43 within the NSC-34 cell line, the levels of total and cytoplasmic Nrf2, as well as its downstream gene NQO1, significantly decreased in a mutant TDP-43 transgenic cell [245]. Treatment of motor neuron-like NSC34 cells overexpressing TDP-43 with diallyl trisulfide, an organosulfur compound in garlic oil with anti-inflammatory properties, activates the Nrf2/ARE pathway, upregulating HO-1 and NQO1 expression, and protects cells from TDP-43-induced damage by scavenging ROS [246]. Sulforaphane enhances the expression of HO-1 and NQO1 in motor neuron-like cells transfected with the wild-type TDP-43 [247]. To validate the significance of NQO1 in the treatment of ALS, comprehensive human studies are essential. These studies may involve retrospective analyses of existing patient data and implementation of prospective clinical trials to evaluate potential therapeutic approaches.

### NQO1 and brain tumors

ROS-sensitive cell signaling pathways play a vital role in the survival, proliferation, and differentiation of tumor cells in various types of brain cancers. These pathways significantly modulate the metabolism, inflammatory processes, and angiogenesis accompanied by brain tumors [248]. Investigations conducted on human glioblastoma (GBM) involving ten patients, as well as primary brain tissue cell cultures and human neuroblastoma (SH-SY5Y) cells, have revealed that the regulation of the Nrf2 pathway and the expression of downstream antioxidant enzymes, such as NQO1, HO-1, and GST, are crucial in the regulation of ROS activity, detoxification of xenobiotics, and inhibition of brain cancer progression [50, 249, 250]. NQO1 contributes to various dimensions of tumorigenesis and treatment response, particularly via the regulation of inflammatory processes. The effect of the ROS retractor C/EBPβ on NQO1 expression in GBM has been evaluated in human glioblastoma cell lines and within an in vivo xenograft animal model. It has been reported that C/EBPβ can counteract ROS in GBM by activating NQO1. This activation of NQO1 exerted an anti-oxidative stress response and reduced the detrimental effects of ROS on GBM cells [44]. Under physiological states, the nuclear-restricted protein (NRP/B) in the brain is typically localized within the nucleus. NRP/B together with Nrf2 plays a crucial role in the protection of human GBM cells against oxidative stress-induced damage [50]. When exposed to oxidative stress, Nrf2 and NQO1 work together to prevent the detrimental effects of oxidative stress. However, in primary brain tumors, NRP/B relocates from the nucleus to the cytoplasm. This cytoplasmic localization of NRP/B alters the function of Nrf2, consequently affecting the transcription and function of downstream genes, including NQO1. The relocation of NRP/B and the alteration of Nrf2-NQO1 function diminish the protective effect against oxidative stress, which is potentially contributed to brain tumorigenesis [50, 249, 251].

NQO1 plays a dual and contradictory role as an anticancer enzyme and an oncogene in the evolution of GBM. Overexpressing NQO1 significantly enhances the cell growth of U87MG and LN229 GBM cells. Conversely, depletion of NQO1 significantly reduces cell proliferation, suggesting the critical role of NQO1 values in determining the proliferation of these tumor cells [252]. NQO1, acting as a downstream target gene of phosphatase and tensin homolog, can effectively reduce ROS values in GBM and facilitate tumorigenesis [252]. Relevant studies demonstrated that the inactivation of glutathione-S-transferase Pi 1 (GSTP1) and NQO1 amplifies ROS-induced tissue damage and leads to the induction of apoptosis and consequently to the inhibition of U87MG GBM cell proliferation. Thus, inhibition of GSTP1 and NQO1 is suggested as a new treatment strategy for GBM [253]. Furthermore, the expression levels of NQO1 can impact the efficacy of tumor treatment in specific types of brain tumors. A correlation has been described between high levels of NQO1 expression and increased radiation resistance in primary mouse models of diffuse intrinsic pontine glioma. The intrinsic radiosensitivity of brainstem gliomas was governed by the p53 signaling pathway, which was linked to the suppression of the Nrf2 pathway gene *NQO1*. The deletion of p53 led to the upregulation of NQO1 expression and a substantial enhancement in tumor resistance to radiation therapy in vivo. The modulation of the elevated levels of NQO1 in tumors suggests a potential method to enhance the efficacy of radiotherapy in brainstem gliomas [254]. NQO1 expression value has the potential to serve as a prognostic biomarker in GBM. Using a dataset with 31 samples derived from the Cancer Genome Atlas database, a positive correlation has been revealed between NQO1 expression and the degree of malignancy in GBM tissues. Modulating NQO1 levels can potentially induce anticancer effects through a non-apoptotic necrosis mechanism in U87MG and U251 glioma cells [255]. NQO1, an antioxidant factor and a protector of p53, plays a crucial role in protecting cells against oxidative stress and DNA damage in head and neck tumors [256]. Alterations in NQO1 function and/or expression can disrupt the equilibrium of cell death pathways, potentially resulting in NQO1-dependent necrosis as a cellular demise mechanism [3, 257, 258]. The administration of temozolomide, an alkylating agent commonly applied for the treatment of GBM, can interact with NQO1 and lead to non-apoptotic necrosis-induced cell death in human U251MG and U87MG GBM cells [259]. Various other potential antitumor compounds, including 2-methoxy-6-acetyl-7-methyljuglone [255], chlorpyrifos (an organophosphate pesticide) [260], and FTY720 (a sphingosine-1-phosphate receptor modulator) [259], have been found to exert cytotoxic effects on various GBM cancer cells by targeting NQO1. Multiple studies have demonstrated that individuals with glioma carrying the isocitrate dehydrogenase 1 (IDH1) R132H mutation show greater sensitivity to the treatment with temozolomide [261]. The IDH1 mutation can lead to a decrease in NADPH values and the accumulation of ROS in GBM cells. Interestingly, when IDH1 R132H cells were exposed to temozolomide, a reduction in NQO1 expression has been observed. This observation suggests a potential involvement of NQO1 in the resistance mechanism of GBM cells to chemotherapy [262]. Studies on fourteen primary and recurrent GBM specimens obtained from patients receiving temozolomide and radiation therapy demonstrated a correlation between the nuclear Nrf2 levels and the time to GBM recurrence. The combined treatment of temozolomide and ionizing radiation has been shown to activate the Nrf2-ARE pathway in human GBM cells. This activation leads to a significant increase in the expression of NQO1 and HO-1, which suggests their involvement in the cellular response to this treatment regimen [263]. Investigations on four different GBM cell lines have shown that the diminished activity of NQO1 is a possible mechanism of resistance to heat shock protein 90 inhibitors in GBM [59]. Pharmacological chaperones offer promising prospects for rectifying the misfolding of NQO1 and enhancing enzyme stability and function. This approach could play a therapeutic role in diverse neurological diseases, including brain tumors [72]. The utilization of a small molecular chaperone exhibited the ability to restore the native wild-type conformation of NQO1 and a substantial enhancement in the enzymatic activity of the P187S variant protein [264]. Furthermore, by using four glioma cell lines (U251, T98G, LN-229, and A172), it has been shown that the interplay between NQO1 and the anti-oncogenic miR-1321 with the oncogene serpin family A member 1 has been shown to impact the proliferation and apoptosis of glioma cells [265]. Targeting the NQO1-mediated pathway of ferroptosis represents another novel therapeutic strategy for the treatment of glioma [266]. Reducing ferroptosis by suppressing KEAP1, which leads to the nuclear translocation of Nrf2 and enhanced expression of NQO1 and HO-1, has demonstrated significant inhibition of U87 GBM cell xenograft growth in the brain ventricles of nude mice [267]. Moreover, NQO1 shows potential as a diagnostic marker for different types of tumors. Theranostic prodrugs based on NQO1 allow for the monitoring of quinone moiety reduction and release of the parent drug, while also showing selective cytotoxicity against cancer cells [268]. Future studies on the role of NQO1 in the pathogenesis of brain tumors can investigate the interactions of NQO1 with various brain tumorigenesis pathways, personalized therapies based on NQO1 levels and genetic profiles, feedback mechanisms regulating NQO1 in brain tumors, and the factors triggering the transition of NQO1 from a protective to a detrimental role in GBM.

## Developing novel therapeutic and/or diagnostic compounds by targeting NQO1

In the past few years, significant progress has been made in uncovering the pathological roles of NQO1 in various neurological disorders. Exogenous compounds that act as modulators of NQO1 have been extensively studied. However, the identification of endogenous substances that modulate this enzyme necessitates further in-depth investigations. The different roles of NQO1 in the CNS present a significant challenge in the selective modulation of a single isolated function by novel compounds [3]. Various substances that have been developed to regulate the NQO1 enzyme could also interact with other enzymes or proteins, such as cytochrome P450 reductase [269]. Efforts are underway to design compounds that selectively target NQO1 without interacting with other crucial cellular processes or proteins and minimize off-target effects [270, 271]. Integrating a feedback mechanism or regulating enzyme expression may reduce potential off-target effects. Considering the functions of NQO1 in reducing oxidative stress through various mechanisms, such as acting as a redox switch and influencing cellular damage and death by generating NAD^+^, it is valuable to consider further efforts in the development of novel compounds to fight against neuroinflammation, neurodegeneration, and the progression of brain malignancies. Nanotechnology has emerged as a novel approach to enhance the delivery of bioactivatable substances targeting NQO1 to specific cell types [272]. Using nano-carriers, such as nano-particles, liposomes, or amphiphilic polymers, can promote the stability, solubility, bioavailability, and ability to penetrate the blood–brain barrier of NQO1-targeted drugs [273]. One of the major challenges in using NQO1-responsive pro-drugs and nano-carriers is the possibility of unintended activation by other reductase enzymes, such as P450 reductase and NADPH [274]. The cross-reactivity of these enzymes can result in off-target effects and compromise the specificity of drug delivery. Cutting-edge techniques, such as the enzyme-responsive nano-drug delivery system, offer a valuable approach for fabricating nano-carriers that exhibit precise targeting and reductive release [275]. The integration of multiple activities within a single molecular entity has been used to design stable and potent peptide inhibitors of the Nrf2-KEAP1 pathway with optimal cellular uptake and resistance against degradation in human serum [276]. Investigating the long-term stability and biodegradability of the nano-carriers would be essential from both environmental and systemic health standpoints [277]. Furthermore, due to selectivity, potency, pharmacokinetic properties, and the ready availability of diagnostics for assessing NQO1 in patients, some NQO1 substrates such as deoxynyboquinone, have considerable potential as personalized medicines for the treatment of various disorders, such as brain tumors [278].

Using the enzymatic characteristics of NQO1, a variety of NQO1-activated optical probes for imaging different cells in various tissues, including the brain, have been developed [279]. This capability could be used for detecting early alterations in neuronal tissues and serve as a promising diagnostic tool in neurological disorders [274, 280]. Indeed, fluorescent probes specifically designed for cellular and intra-vital imaging of mitochondrial NQO1 have shown remarkable binding affinity to NQO1. These probes have been successfully used to differentiate between different NQO1-expressing cancer cells and normal cells [274]. Moreover, they have provided valuable insights into the decreased NQO1 activity observed in a cellular model of PD [274]. Fluorescent probes have also been developed to enable non-invasive monitoring of endogenous NQO1 activity in brain tumor cells both in vitro and in transplanted tumors in nude mice [281]. An NQO-1-activated near-infrared agent has been designed to rich into mitochondria and lysosomes and act as a chemotherapeutic compound [282]. A genetically encoded sensor-based metabolic screening tool has been also developed for tracking intracellular NQO1-activated redox cycling [283].

## Conclusion

NQO1 plays a regulatory role in the oxidant/antioxidant equilibrium within the brain by facilitating the detoxification of harmful compounds, protecting cells from oxidative stress, regenerating antioxidants, and its ability to directly interact with other molecules [3, 269, 284]. Available data strongly suggest that targeting NQO1 could be a promising strategy for developing new diagnostic and therapeutic compounds for neurological disorders. Furthermore, NQO1 can be utilized as a biomarker for tracking subtle changes in neural tissues and serve as an early warning system for the emergence of neurological diseases. To develop suitable compounds for targeting NQO1, additional experimental and clinical studies are required to improve our understanding of the basic molecular mechanisms of NQO1 and its particular role in the evolution and progression of neurological disorders. When considering the potential contributions of NQO1 to personalized medicine for neurological disorders, evaluation of its potential interactions with other neurological biomarkers, and using artificial intelligence and big data for a deeper understanding of the complex role of NQO1 in neurological disorders may open up exciting research paths for the future. Acknowledging potential off-target effects, specific conditions where NQO1 may not be a reliable biomarker, and the challenges of translating laboratory discoveries to clinical practice provide a well-rounded and balanced perspective on the role of NQO1 in the field of personalized medicine. Furthermore, the significance lies in the adoption of replicable research designs and methodologies across diverse populations. It is important to note that potential off-target effects, specific conditions in which NQO1 may not function as a reliable biomarker, and the difficulties in translating bench side findings to the bedside represent significant challenges for future studies. Furthermore, the potential different roles of NQO1 in acute and chronic neurological disorders should be considered in future studies.

## Data Availability

The data presented in this study are available on request from the corresponding author.

## Abbreviations

ATP: Adenosine triphosphate
AD: Alzheimer´s disease
ALS: Amyotrophic lateral sclerosis
Aβ: Amyloid-β
AMPK: AMP-activated protein kinase
AP-1: Activator protein 1
ARE: Antioxidant response element enhancer sequence
BDNF: Brain-derived neurotrophic
CNS: Central nervous system
CREB: CAMP response element-binding protein
DA: Dopamine
EAE: Experimental autoimmune encephalomyelitis mouse model
FAD: Flavin adenine dinucleotide
GST: Glutathione S-transferase
GBM: Glioblastoma
GSTP1: Glutathione-S-transferase Pi 1
HIF-1α: Hypoxia-inducible factor 1-alpha
HO-1: Heme oxygenase-1
IDH1: Isocitrate dehydrogenase 1
KEAP1: Kelch-like ECH-associated protein1
MCAO: Middle cerebral artery occlusion
MS: Multiple sclerosis
NQO1: NAD(P)H Quinone Dehydrogenase 1
Nrf2: Nuclear factor erythroid 2-related factor 2
NRP/B: Nuclear-restricted protein
PD: Parkinson's disease
PI3K: Phosphoinositide 3-kinase
RIPK1: Receptor-interacting serine/threonine-protein kinase 1
ROS: Oxidative stresses
SAH: Subarachnoid hemorrhage
SN: Substantia nigra
SOD1: Superoxide dismutase 1
TBI: Traumatic brain injury
TDP-43: Transactive response DNA-binding protein 43
